# Regulated intratumoral expression of IL-12 as a basis for combination therapy in melanoma

**DOI:** 10.1186/1479-5876-12-S1-O11

**Published:** 2014-05-06

**Authors:** John J Nemunaitis, Gerald P Linette, Omid Hamid, Sanjiv S Agarwala, Alexander Starodub, Lei Sun, Francois Lebel, John A Barrett, Jonathan Lewis

**Affiliations:** 1 Mary Crowley Cancer Research Center, Dallas Tx, USA; 2 Washington University, Saint Louis, MO, USA; 3 The Angeles Clinic and Research Institute, Los Angeles, CA, USA; 4 Saint Luke's University Health System, Bethlehem, PA, USA; 5 Indiana University Health Goshen Center for Cancer Care; IN, USA; 6 ZIOPHARM Oncology Inc., Boston, MA, USA

## Background

Major obstacles for the development of immunotherapeutics are the ability of tumors to escape the immune system coupled with toxicity associated with systemic administration. To overcome these challenges, we have developed an adenoviral vector, Ad-RTS-IL-12 (AD), administered intratumorally under control of the RheoSwitch Therapeutic System^®^ gene expression platform. Expression of IL-12 mRNA and IL-12 protein is tightly regulated by the oral administration of a small molecule activator ligand, veledimex (AL).

## Materials and methods

In preclinical studies the antitumor activity of Ad-RTS-mIL-12 in combination with AL was studied in the mouse B16F0 melanoma model. Melanoma (B16F0) cells were inoculated subcutaneously into the right and left flanks of 6-8 week old female C57BL/6 mice. When tumors reached 70-100 mm^3^, animals received a single intratumoral (IT) injection of Ad-RTS-mIL-12 1 x 10^10^ viral particles into the right flank plus AL at a dose of ~200 mg/m^2^ in the chow *ad lib* for the duration of the study. Tumor growth (TG), tumor cytokines and infiltrating T cells were studied. In a human Phase I, 3+3 dose escalation study, subjects with nonresectable stage III/IV melanoma were injected Ad-RTS-hIL-12 IT with a constant dose of 1 x 10^12^ viral particles on the first day of each 21-day cycle, and escalating oral doses of AL (5, 20, 100, and 160 mg) on Days 1-7 of each cycle. In the expanded Phase II portion 8 subjects have been treated with 160 mg.

## Results

In mice, increase in local expression of IL-12 with increasing AL dose resulted in decrease in TG in both the treated and untreated tumors coupled with an increase in tumor infiltrating lymphocytes (TILs) as well as demonstration of systemic immunity. In human subjects, increase in tumor IL-12 mRNA expression and increases TILs (CD8^+^, CD45RO^+^) were observed following treatment of Ad-RTS-IL-12 + AL (100 or 160 mg). Clinical activity without significant toxicity correlated with the highest serum levels of IL-12 which induced interferon-gamma (IFN-γ). Based on these results, preclinical combination studies with signal transduction agents and immunotherapeutics are ongoing and will be presented.

## Conclusions

Delivery of IL-12 intratumorally via an adenoviral vector using RheoSwitch® technology enables finely-controlled expression of IL-12, which is well tolerated and results in an increase in TILs concomitant with a reduction in TG. These findings form the basis for ongoing combination studies in melanoma.

